# Latent and incubation periods of Delta, BA.1, and BA.2 variant cases and associated factors: a cross-sectional study in China

**DOI:** 10.1186/s12879-024-09158-7

**Published:** 2024-03-06

**Authors:** Yu Li, Xinli Jiang, Yan Qiu, Feng Gao, Hualei Xin, Dan Li, Ying Qin, Zhongjie Li

**Affiliations:** 1 Division of Infectious Disease Control and Prevention, Chinese Center for Disease Control and Prevention, Beijing, 102206 China; 2 WHO Collaborating Centre for Infectious Disease Epidemiology and Control, School of Public Health, Li Ka Shing Faculty of Medicine, The University of Hong Kong, Hong Kong Special Administrative Region, Hong Kong, China; 3 School of Population Medicine and Public Health, Chinese Academy of Medical Science (CAMS), Peking Union Medical College (PUMC), No. 9, Dongdan Santiao, Dongcheng District, Beijing, 100730 China

**Keywords:** SARS-CoV-2, Delta, BA.1, BA.2, Latent period, Incubation period, Associated factor

## Abstract

**Background:**

The latent and incubation periods characterize the transmission of infectious viruses and are the basis for the development of outbreak prevention and control strategies. However, systematic studies on the latent period and associated factors with the incubation period for SAS-CoV-2 variants are still lacking. We inferred the two durations of Delta, BA.1, and BA.2 cases and analyzed the associated factors.

**Methods:**

The Delta, BA.1, and BA.2 (and its lineages BA.2.2 and BA.2.76) cases with clear transmission chains and infectors from 10 local SAS-CoV-2 epidemics in China were enrolled. The latent and incubation periods were fitted by the Gamma distribution, and associated factors were analyzed using the accelerated failure time model.

**Results:**

The mean latent period for 672 Delta, 208 BA.1, and 677 BA.2 cases was 4.40 (95%CI: 4.24 ~ 4.63), 2.50 (95%CI: 2.27 ~ 2.76), and 2.58 (95%CI: 2.48 ~ 2.69) days, respectively, with 85.65% (95%CI: 83.40 ~ 87.77%), 97.80% (95%CI: 96.35 ~ 98.89%), and 98.87% (95%CI: 98.40 ~ 99.27%) of them starting to shed viruses within 7 days after exposure. In 405 Delta, 75 BA.1, and 345 BA.2 symptomatic cases, the mean latent period was 0.76, 1.07, and 0.79 days shorter than the mean incubation period [5.04 (95%CI: 4.83 ~ 5.33), 3.42 (95%CI: 3.00 ~ 3.89), and 3.39 (95%CI: 3.24 ~ 3.55) days], respectively. No significant difference was observed in the two durations between BA.1 and BA.2 cases. After controlling for the sex, clinical severity, vaccination history, number of infectors, the length of exposure window and shedding window, the latent period [Delta: exp(β) = 0.81, 95%CI: 0.66 ~ 0.98, *p* = 0.034; Omicron: exp(β) = 0.82, 95%CI: 0.71 ~ 0.94, *p* = 0.004] and incubation period [Delta: exp(β) = 0.69, 95%CI: 0.55 ~ 0.86, *p* < 0.001; Omicron: exp(β) = 0.83, 95%CI: 0.72 ~ 0.96, *p* = 0.013] were significantly shorter in 18 ~ 49 years but did not change significantly in ≥ 50 years compared with 0 ~ 17 years.

**Conclusion:**

Pre-symptomatic transmission can occur in Delta, BA.1, and BA.2 cases. The latent and incubation periods between BA.1 and BA.2 were similar but shorter compared with Delta. Age may be associated with the latent and incubation periods of SARS-CoV-2.

**Supplementary Information:**

The online version contains supplementary material available at 10.1186/s12879-024-09158-7.

## Background

Studies in the early COVID-19 pandemic have confirmed that viral genomes and live viruses are detectable in the upper respiratory tract before the symptoms onset in SARS-CoV-2 cases [1]. The incubation period is the time interval from infection to symptom onset [2]. Some scholars define the latent period of an infectious disease as the time interval from infection to becoming infectious [3–5]. Since the latter was difficult to determine, the time when the virus was first detected in the host samples is usually used as a substitute [2, 4, 5]. Therefore, assessing whether the latent period is shorter than the incubation period can indicate whether pre-symptomatic transmission can occur. However, systematic studies on the latent period are still lacking.

In addition, systematic studies on factors that impact the latent and incubation periods of SARS-CoV-2 variants remain scanty. Several studies based on small samples and univariate analysis of the early stages of the pandemic suggested that the incubation period of SARS-CoV-2 might be associated with age [6–9] and clinical severity [10–12]. A case series analysis from France using multivariable linear regression to identify factors associated with the duration of the incubation period did not cover participants aged < 18 years and Omicron sublineages [13]. Furthermore, with the increasing immune escape capacity of SARS-CoV-2 variants [14–16], evidence of whether vaccination alters latent and incubation periods is needed.

In this study, we estimated the latent and incubation periods and explored the individual factors associated with these durations using a large number of patients infected with Delta, BA.1, and BA.2.

## Methods

### Data collection

We selected reverse transcriptase PCR (RT-PCR) confirmed SARS-CoV-2 infected individuals with clear transmission chains and infectors from the epidemiological survey reports obtained from four local Delta epidemics in four cities (Guangzhou of Guangdong, Ruili of Yunnan, Yangzhou of Jiangsu, and Nanjing of Jiangsu) and six local Omicron epidemics in five cities (Anyang of Henan, Baise of Guangxi, Jilin of Jilin, Yibin of Sichuan, and Beijing) in China during May 1, 2021 and September 30, 2022. The epidemiological survey report provided a detailed description on the basic information (e.g., age, sex, comorbidity), epidemiological timelines (e.g., potential time of infection, potential infectors, symptom onset, laboratory confirmation) and exposure information of SARS-CoV-2 infected individual if known [2]. Infected individuals with clear transmission chains and infectors meant they could identify at least one possible infector, which had exposure before the infected individuals exposed to them. In our study, infected individuals’ exposure to the infectors involved one or more of the following ways: (i) living together; (ii) medical care; (iii) meals together; (iv) working together; (v) studying together; (vi) recreation in the same room; (vii) daily conversation; (viii) transport together; (ix) room only, no direct contact communication.

To estimate the latent period for each infected individual, we extracted the first and latest dates of exposure (exposure window: $$ {E}_{L}$$~$$ {E}_{U}$$) to infectors. Dates of the last negative RT-PCR test sampling and the first positive RT-PCR test sampling (shedding window: $$ {V}_{L}\sim{V}_{U}$$), which provide the time at which virus shedding began, was also extracted from the epidemiological survey reports. We also obtained dates of symptom onset for symptomatic cases to estimate the incubation period. Asymptomatic cases and symptomatic cases whose date of symptom onset was unclear were used only for estimating and analyzing the latent period. Other information, including age, sex, clinical severity, vaccination history, comorbidity, number of infectors, the length of exposure window ($$ {E}_{U}$$-$$ {E}_{L}$$) and shedding window ($$ {V}_{U}$$-$$ {V}_{L}$$), was also extracted from epidemiological survey reports to analyze factors of the latent and incubation the periods. Cases’ clinical severity was categorized as asymptomatic, mild, moderate, severe, and critical (**Additional file 1**). Vaccination history, including vaccine type, the dose of vaccination, and date of the dose, was collected for each case. Cases were defined as unvaccinated, partially vaccinated, fully vaccinated, and booster vaccination (**Additional file 1**).

### Statistical analysis

Characteristics of SARS-CoV-2 cases were presented with frequencies and percentages. The durations of exposure window and shedding window were reported as median and interquartile range (IQR). Differences in the composition ratio between Delta and Omicron cases by basic characteristics were analyzed using the chi-square test.

Different from the unilateral interval-censored data of the incubation period (exposure window and the exact date for the symptom onset), there was doubly censored data for the latent period (exposure window and shedding window). Parametric models, including Gamma, Lognormal, and Weibull distributions, using maximum likelihood estimation were fitted to the interval-censoring data (**Additional file 1**). We selected the best-fitting distribution as the one with the lowest Akaike information criterion (AIC) score (**Additional file 2: Table ****S1**). A parametric bootstrap approach with 1000 resamples was used to obtain 95% confidence intervals for each parameter [4]. 

SARS-CoV-2 infected individuals may shed virus during the incubation period [1, 17–20]. Furthermore, previous studies based on single-site exposure have shown that the minimum incubation period was between 0 and 1 day after exposure to infectors with the Delta variant [5] and 1 day in those infected with the Omicron variant, [21] suggesting that the earliest time of virus shedding in such circumstances was within 1 day after exposure. Therefore, for cases where the last negative sampling date ($$ {V}_{L}$$) was unclear or before the date of first exposure, we uniformly used the date of $$ {E}_{L}$$+0.5 days as a lower limit of shedding window. In the sensitivity analysis, we also showed the results of latent period estimation using $$ {E}_{L}$$+0 and $$ {E}_{L}$$+0.9 as substitutes for $$ {V}_{L}$$ in the above cases(**Additional file 2: Table ****S2**).

A linear regression model, the accelerated failure time model (AFT), with maximum likelihood estimation was used to explore the possible relationship between selected factors and the latent as well as the incubation periods (**Additional file 1**). Sex, age, vaccination history, clinical severity, number of infectors, and exposure window were included in the multivariate analyses following univariate analyses. The AFT model was also used to test the significance of the difference between the latent period and the incubation period (**Additional file 1**). Parametric regression models for interval-censored data may be applied in R (through the package “icenReg”).

All analyses were performed using R software version 4.0.2 (R Foundation for Statistical Computing, Vienna, Austria). All P value < 0.05 (two-sided) was considered significant.

## Results

### Characteristics of participants

A total of 672 Delta, 208 BA.1, and 677 BA.2 (and its lineage BA.2.2 and BA.2.76) cases were included, 420 (62.50%), 75 (36.06%), and 345 (50.96%) of which had a clear date of symptom onset, respectively. The median age (*IQR*) of cases infected with Delta and Omicron was 49 (32 ~ 65) years and 29 (20 ~ 48) years. There were more males in the Omicron group than in the Delta group. A total of 22 cases were severe or critical, of which 20 (90.91%) were aged ≥ 70 years and 21 (95.45%) were infected with the Delta variant. No case in the Delta group had received a booster vaccination. Among those who were at least fully vaccinated in the Delta and Omicron groups, 91.18% (186/204) and 98.98% (777/785) of them, respectively, had received inactivated vaccines. The median exposure window (*IQR*) was 4 (1 ~ 8) days and 2 (1~5) days for the Delta and Omicron groups. The median shedding window (*IQR*) for Delta and Omicron variants was 3 (2~6) days and 2 (1~3) days, respectively. Details were shown in Table 1.

**Table 1:** Characteristics of individuals infected with SARS-CoV-2 enrolled in study

	All cases for latent period (%)				Symptomatic cases for incubation period (%)		
	Delta	Omicron	p value		Delta	Omicron	p value
**All**	672 (100)	885 (100)	··		405 (100)	420 (100)	··
**Sex**			< 0.001				< 0.001
Male	266 (39.6)	459 (51.9)	··		148 (36.5)	206 (49.0)	··
Female	406 (60.4)	426 (48.1)	··		257 (63.5)	214 (51.0)	··
**Age**			< 0.001				< 0.001
0 ~ 17	96 (14.3)	147 (16.6)	··		43 (10.6)	79 (18.8)	··
18 ~ 49	248 (36.9)	534 (60.3)	··		172 (42.5)	266 (63.3)	··
50~	328 (48.8)	204 (23.1)	··		190 (46.9)	75 (17.9)	··
**Comorbidity**			< 0.001				< 0.001
No	293 (43.6)	193 (21.8)	··		181 (44.7)	96 (22.8)	··
Yes	234 (34.8)	63 (7.1)	··		135 (33.3)	31 (7.4)	··
Unknown	145 (21.6)	629 (71.1)	··		89 (22.0)	293 (69.8)	··
**Clinical severity**			< 0.001				< 0.001
Asymptomatic	12 (1.79)	154 (17.4)	··				··
Mild	128 (19.0)	615 (69.5)	··		64 (15.8)	371 (88.3)	··
Moderate	511 (76.0)	115 (13.0)	··		328 (81.0)	48 (11.4)	··
Severe/Critical	21 (3.1)	1 (0.1)	··		13 (3.2)	1 (0.2)	··
**Vaccination history**			< 0.001				< 0.001
Unvaccinated	292 (43.4)	67 (7.6)	··		169 (41.7)	38 (9.0)	··
Partially vaccinated	176 (26.2)	33 (3.7)	··		111 (27.4)	20 (4.8)	··
Fully vaccinated	204 (30.4)	362 (40.9)	··		125 (30.9)	164 (39.0)	··
Booster	0 (0.0)	423 (47.8)	··		0 (0.0)	198 (47.1)	··
**Number of infectors**			< 0.001				< 0.001
> 1	191 (28.4)	490 (55.4)	··		119 (29.4)	211 (50.2)	··
= 1	481 (71.6)	395 (44.6)	··		286 (70.6)	209 (49.8)	··
**Exposure window (median**, ***IQR*)**	4 (1 ~ 8) days	2 (1 ~ 5) days	··		3 (1 ~ 8) days	2 (1 ~ 5) days	··
**Shedding window (median**, ***IQR*)**	3 (2 ~ 6) days	2 (1 ~ 3) days	··		3 (2 ~ 6) days	2 (1 ~ 3) days	··

*Notes* Differences in the composition ratio between Delta and Omicron cases were analyzed by chi-square test. IQR represented the interquartile range

### Parameter estimation

The Gamma, lognormal, and Weibull parametric models were fitted for the distributions of latent and incubation periods. Gamma distribution had a smaller AIC score (**Additional file 2: Table ****S1**).

**Fig. 1:**
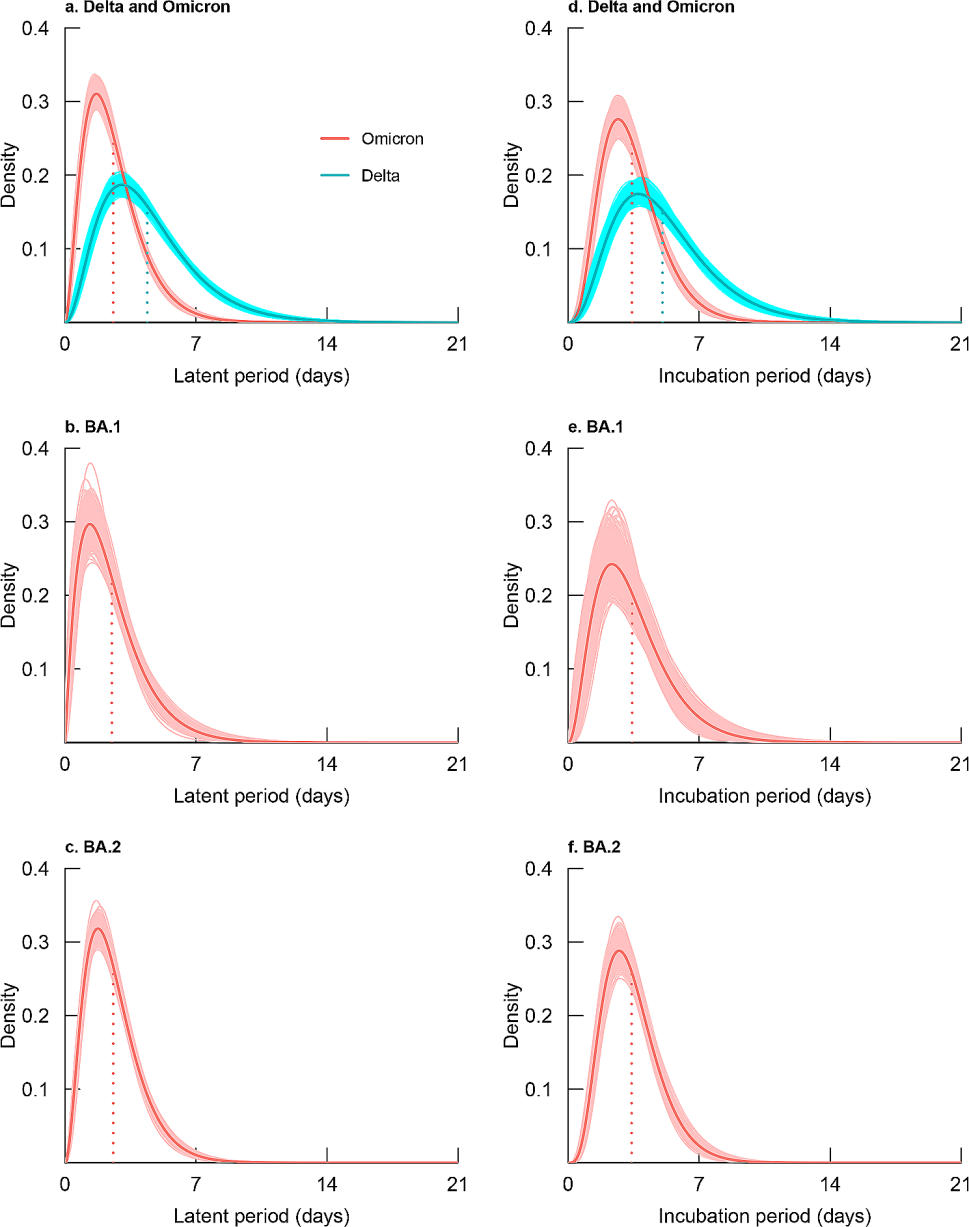
Probability density distribution of latent and incubation periods for SARS-CoV-2 cases. Legends: Shaded parts represented the 95% confidence interval. The intersection points of the dashed lines and the horizontal axes were the mean values of latent periods or incubation periods

The mean latent period for Delta, BA.1, and BA.2 was 4.40 (95%CI: 4.24 ~ 4.63) days, 2.50 (95%CI: 2.27 ~ 2.76) days, and 2.58 (95%CI: 2.48 ~ 2.69) days, respectively. 85.65% (95%CI: 83.40 ~ 87.77%), 97.80% (95%CI: 96.35 ~ 98.89%), and 98.87% (95%CI: 98.40 ~ 99.27%) of Delta, BA.1, and BA.2 cases started to shed the virus within 7 days after exposure (Fig. 1). For sensitivity analyses of latent period estimates, the three methods yielded similar means, standard deviations, 95th percentile, and 99th percentile in both Omicron and Delta cases (**Additional file 2: Table ****S2**).

The mean incubation period of symptomatic cases infected with Delta, BA.1, and BA.2 variants was 5.04 (95%CI: 4.83 ~ 5.33) days, 3.42 (95%CI: 3.00 ~ 3.89) days, and 3.39 (95%CI: 3.24 ~ 3.55) days. For 98% of Delta and Omicron cases, symptoms appeared within 11.60 (95%CI: 10.90 ~ 12.42) days and 7.41 (95%CI: 6.98 ~ 7.90) days after exposure (Table 2; Fig. 1).

**Table 2:** Parametric estimates of latent and incubation periods of SARS-CoV-2 using Gamma distribution

	Latent period (days)					Incubation period (days)			
	Delta (672)	Omicron (885)	BA.1 (208)	BA.2 (677)		Delta (405)	Omicron (420)	BA.1 (75)	BA.2 (345)
Mean	4.40 (4.24 ~ 4.63*)	2.58 (2.48 ~ 2.68)	2.50 (2.27 ~ 2.76)	2.58 (2.48 ~ 2.69)		5.04 (4.83 ~ 5.33)	3.41 (3.27 ~ 3.58)	3.42 (3.00 ~ 3.89)	3.39 (3.24 ~ 3.55)
Standard deviation	2.46 (2.32 ~ 2.65)	1.52 (1.43 ~ 1.61)	1.71 (1.48 ~ 1.95)	1.46 (1.36 ~ 1.56)		2.57 (2.37 ~ 2.79)	1.59 (1.47 ~ 1.72)	1.92 (1.54 ~ 2.34)	1.51 (1.38 ~ 1.65)
Median	3.95 (3.81 ~ 4.16)	2.28 (2.19 ~ 2.38)	2.12 (1.92 ~ 2.34)	2.32 (2.22 ~ 2.42)		4.61 (4.41 ~ 4.87)	3.17 (3.04 ~ 3.32)	3.07 (2.66 ~ 3.50)	3.17 (3.03 ~ 3.33)
25th percentile	2.59 (2.47 ~ 2.77)	1.46 (1.38 ~ 1.54)	1.24 (1.09 ~ 1.42)	1.51 (1.43 ~ 1.60)		3.16 (2.98 ~ 3.38)	2.25 (2.13 ~ 2.38)	2.01 (1.68 ~ 2.38)	2.29 (2.16 ~ 2.43)
75th percentile	5.72 (5.50 ~ 6.02)	3.38 (3.25 ~ 3.52)	3.35 (3.04 ~ 3.70)	3.37 (3.22 ~ 3.51)		6.46 (6.18 ~ 6.82)	4.31 (4.12 ~ 4.53)	4.45 (3.90 ~ 5.04)	4.25 (4.06 ~ 4.46)
95th percentile	9.07 (8.95 ~ 9.61)	5.48 (5.22 ~ 5.74)	5.81 (5.20 ~ 6.48)	5.36 (5.08 ~ 6.64)		9.87 (9.34 ~ 10.54)	6.38 (6.04 ~ 6.77)	7.06 (6.00 ~ 8.29)	6.20 (5.83 ~ 6.57)
97th percentile	10.04 (9.55 ~ 10.67)	6.09 (5.79 ~ 6.39)	6.54 (5.83 ~ 7.34)	5.93 (5.60 ~ 6.26)		10.85 (10.22 ~ 11.60)	6.97 (6.57 ~ 7.40)	7.82 (6.58 ~ 9.25)	6.74 (6.33 ~ 7.17)
98th percentile	10.79 (10.25 ~ 11.48)	6.57 (6.23 ~ 6.89)	7.11 (6.30 ~ 8.01)	6.38 (6.02 ~ 6.74)		11.60 (10.90 ~ 12.42)	7.41 (6.98 ~ 7.90)	8.40 (7.02 ~ 9.97)	7.16 (6.71 ~ 7.64)
99th percentile	12.03 (11.41 ~ 12.83)	7.36 (6.97 ~ 7.75)	8.07 (7.08 ~ 9.14)	7.12 (6.70 ~ 7.55)		12.84 (12.02 ~ 13.79)	8.16 (7.65 ~ 8.71)	9.38 (7.79 ~ 11.23)	7.85 (7.33 ~ 8.41)

*Notes* *95% confidence interval

### Analyses of associated factors

After adjusting for gender, age, exposure and shedding windows, clinical severity, number of vaccination doses, and the number of infectors, we found that compared with the Delta, the latent period of the Omicron was significantly shorter by 40% [exp($\beta$) = 0.60, 95%CI: 0.53 ~ 0.67, *p* < 0.001], whereas the incubation period was significantly shorter by 26% [exp($\beta$) = 0.74, 95%CI: 0.65 ~ 0.84, *p* < 0.001] (**Additional file 2: Table ****S3**). However, no statistical significance was observed in the latent and incubation periods between BA.1 and BA.2 (Tables 3 and 4).

**Table 3:** Association between selected factors and latent as well as incubation periods using univariate AFT model

	Latent period						Incubation period				
	Delta (672)			Omicron (885)			Delta (405)			Omicron (420)	
	exp ($\varvec{\beta }$) (95%CI)	p value		exp ($\varvec{\beta }$) (95%CI)	p value		exp ($\varvec{\beta }$) (95%CI)	p value		exp ($\varvec{\beta }$) (95%CI)	p value
**Sex**											
Male	1 (ref)	··		1 (ref)	··		1 (ref)	··		1 (ref)	··
Female	1.02 (0.92 ~ 1.13)	0.735		0.98 (0.89 ~ 1.09)	0.760		1.01 (0.89 ~ 1.13)	0.941		0.98 (0.88 ~ 1.08)	0.633
**Age**											
0 ~ 17	1 (ref)	··		1 (ref)	··		1 (ref)	··		1 (ref)	··
18 ~ 49	0.76 (0.64 ~ 0.91)	0.002		0.74 (0.65 ~ 0.84)	< 0.001		0.68 (0.56 ~ 0.83)	< 0.001		0.80 (0.70 ~ 0.92)	0.002
50~	0.95 (0.81 ~ 1.11)	0.500		1.02 (0.88 ~ 1.18)	0.805		0.86 (0.71 ~ 1.04)	0.128		0.97 (0.81 ~ 1.16)	0.733
**Vaccination history**											
Unvaccinated	1 (ref)	··		1 (ref)	··		1 (ref)	··		1 (ref)	··
Partially vaccinated	0.96 (0.84 ~ 1.09)	0.492		1.02 (0.75 ~ 1.40)	0.900		0.92 (0.80 ~ 1.06)	0.229		0.94 (0.70 ~ 1.25)	0.676
Fully vaccinated	0.91 (0.80 ~ 1.03)	0.121		1.10 (0.90 ~ 1.33)	0.357		0.88 (0.77 ~ 1.01)	0.070		1.05 (0.87 ~ 1.27)	0.584
Booster	··	··		0.97 (0.80 ~ 1.18)	0.762		··	··		1.01 (0.84 ~ 1.22)	0.914
**Clinical severity**											
Asymptomatic	1 (ref)	··		1 (ref)	··		NA	NA		NA	NA
Mild	1.40 (0.92 ~ 2.12)	0.112		1.05 (0.93 ~ 1.19)	0.409		1 (ref)	··		1 (ref)	··
Moderate**/**Severe/Critical	1.30 (0.87 ~ 1.93)	0.204		1.09 (0.92 ~ 1.29)	0.317		0.98 (0.83 ~ 1.16)	0.831		1.06 (0.90 ~ 1.24)	0.489
**Number of infectors**											
> 1	1 (ref)	··		1 (ref)	··		1 (ref)	··		1 (ref)	··
= 1	0.92 (0.82 ~ 1.04)	0.184		1.04 (0.94 ~ 1.14)	0.471		0.92 (0.81 ~ 1.05)	0.229		1.07 (0.97 ~ 1.19)	0.186
**Exposure window**											
**≤** 3 days	1 (ref)	··		1 (ref)	··		1 (ref)	··		1 (ref)	··
> 3 days	1.07 (0.96 ~ 1.19)	0.227		1.54 (1.37 ~ 1.73)	< 0.001		1.05 (0.94 ~ 1.19)	0.388		1.54 (1.38 ~ 1.73)	< 0.001
**Shedding window**											
**≤** 3 days	1 (ref)	··		1 (ref)	··		1 (ref)	··		1 (ref)	··
> 3 days	0.79 (0.70 ~ 0.89)	< 0.001		0.85 (0.70 ~ 1.02)	0.086		0.95 (0.85 ~ 1.06)	0.376		1.21 (1.07 ~ 1.38)	0.002
**Omicron sublineages**											
BA.1	NA	NA		1 (ref)	··		NA	NA		1 (ref)	··
BA.2	NA	NA		0.94 (0.83 ~ 1.05)	0.262		NA	NA		0.98 (0.86 ~ 1.12)	0.759
**Comorbidity**											
No	1 (ref)	··		1 (ref)	··		1 (ref)	··		1 (ref)	··
Yes	1.00 (0.89 ~ 1.12)	0.954		1.10 (0.89 ~ 1.35)	0.364		0.98 (0.86 ~ 1.11)	0.695		1.07 (0.84 ~ 1.35)	0.597

*Notes* NA represented that the figure was not applicable for this cell

**Table 4:** Association between selected factors and latent as well as incubation periods using multivariate AFT model

	Latent period						Incubation period				
	Delta (672)			Omicron (885)			Delta (405)			Omicron (420)	
	exp ($\varvec{\beta }$) (95%CI)	p value		exp ($\varvec{\beta }$) (95%CI)	p value		exp ($\varvec{\beta }$) (95%CI)	p value		exp ($\varvec{\beta }$) (95%CI)	p value
**Sex**											
Male	1 (ref)	··		1 (ref)	··		1 (ref)	··		1 (ref)	··
Female	1.01 (0.91 ~ 1.13)	0.839		0.95 (0.87 ~ 1.05)	0.334		0.99 (0.88 ~ 1.11)	0.887		0.97 (0.88 ~ 1.07)	0.546
**Age**											
0 ~ 17	1 (ref)	··		1 (ref)	··		1 (ref)	··		1 (ref)	··
18 ~ 49	0.81 (0.66 ~ 0.98)	0.034		0.82 (0.71 ~ 0.94)	0.004		0.69 (0.55 ~ 0.86)	< 0.001		0.83 (0.72 ~ 0.96)	0.013
50~	1.04 (0.87 ~ 1.25)	0.669		1.07 (0.92 ~ 1.26)	0.373		0.89 (0.71 ~ 1.10)	0.270		0.95 (0.80 ~ 1.14)	0.593
**Vaccination history**											
Unvaccinated	1 (ref)	··		1 (ref)	··		1 (ref)	··		1 (ref)	··
Partially vaccinated	0.97 (0.85 ~ 1.11)	0.676		1.05 (0.78 ~ 1.41)	0.757		0.96 (0.84 ~ 1.11)	0.606		0.98 (0.75 ~ 1.27)	0.856
Fully vaccinated	0.98 (0.85 ~ 1.13)	0.789		1.07 (0.88 ~ 1.28)	0.501		1.00 (0.86 ~ 1.16)	0.962		1.03 (0.86 ~ 1.23)	0.751
Booster	··	··		0.97 (0.81 ~ 1.17)	0.780		··	··		1.02 (0.85 ~ 1.21)	0.864
**Clinical severity**											
Asymptomatic	1 (ref)	··		1 (ref)	··		NA	NA		NA	NA
Mild	1.35 (0.90 ~ 2.04)	0.148		1.00 (0.89 ~ 1.13)	0.969		1 (ref)	··		1 (ref)	··
Moderate**/**Severe/Critical	1.23 (0.83 ~ 1.83)	0.304		0.99 (0.84 ~ 1.17)	0.914		1.02 (0.86 ~ 1.20)	0.864		1.07 (0.91 ~ 1.26)	0.395
**Number of infectors**											
> 1	1 (ref)	··		1 (ref)	··		1 (ref)	··		1 (ref)	··
= 1	0.92 (0.81 ~ 1.04)	0.190		0.96 (0.87 ~ 1.05)	0.378		0.90 (0.79 ~ 1.03)	0.133		0.99 (0.90 ~ 1.10)	0.904
**Exposure window**											
**≤** 3 days	1 (ref)	··		1 (ref)	··		1 (ref)	··		1 (ref)	··
> 3 days	1.06 (0.94 ~ 1.19)	0.359		1.51 (1.35 ~ 1.70)	< 0.001		1.05 (0.92 ~ 1.19)	0.469		1.48 (1.32 ~ 1.66)	< 0.001
**Shedding window**											
**≤** 3 days	1 (ref)	··		1 (ref)	··		1 (ref)	··		1 (ref)	··
> 3 days	0.80 (0.70 ~ 0.90)	< 0.001		0.85 (0.71 ~ 1.02)	0.081		0.96 (0.86 ~ 1.07)	0.469		1.21 (1.08 ~ 1.36)	0.001
**Omicron sublineages**											
BA.1	NA	NA		1 (ref)	··		NA	NA		1 (ref)	··
BA.2	NA	NA		0.99 (0.88 ~ 1.12)	0.886		NA	NA		1.03 (0.90 ~ 1.18)	0.648

*Notes* NA represented that the figure was not applicable for this cell

Similar to the univariate analyses, the multivariable analyses showed that the latent period [Delta: exp($\beta$) = 0.81, 95%CI: 0.66~0.98, *p* = 0.034; Omicron: exp($\beta$) = 0.82, 95%CI: 0.71~0.94, *p* = 0.004] and incubation period [Delta: exp($\beta$) = 0.69, 95%CI: 0.55~0.86, *p* < 0.001; Omicron: exp($\beta$) = 0.83, 95%*CI*: 0.72~0.96, *p* = 0.013] were significantly shorter for individuals aged 18 ~ 49 years than those aged 0 ~ 17 years. However, no statistically significant difference was found between individuals aged ≥ 50 years and those aged 0~17 years (*p* > 0.05) for both durations. No significant differences were observed in both latent and incubation periods between different genders, vaccination doses, and clinical severity for both Delta and Omicron groups (*p* > 0.05) (Tables 3 and 4).

### Difference between the two durations

**Fig. 2:**
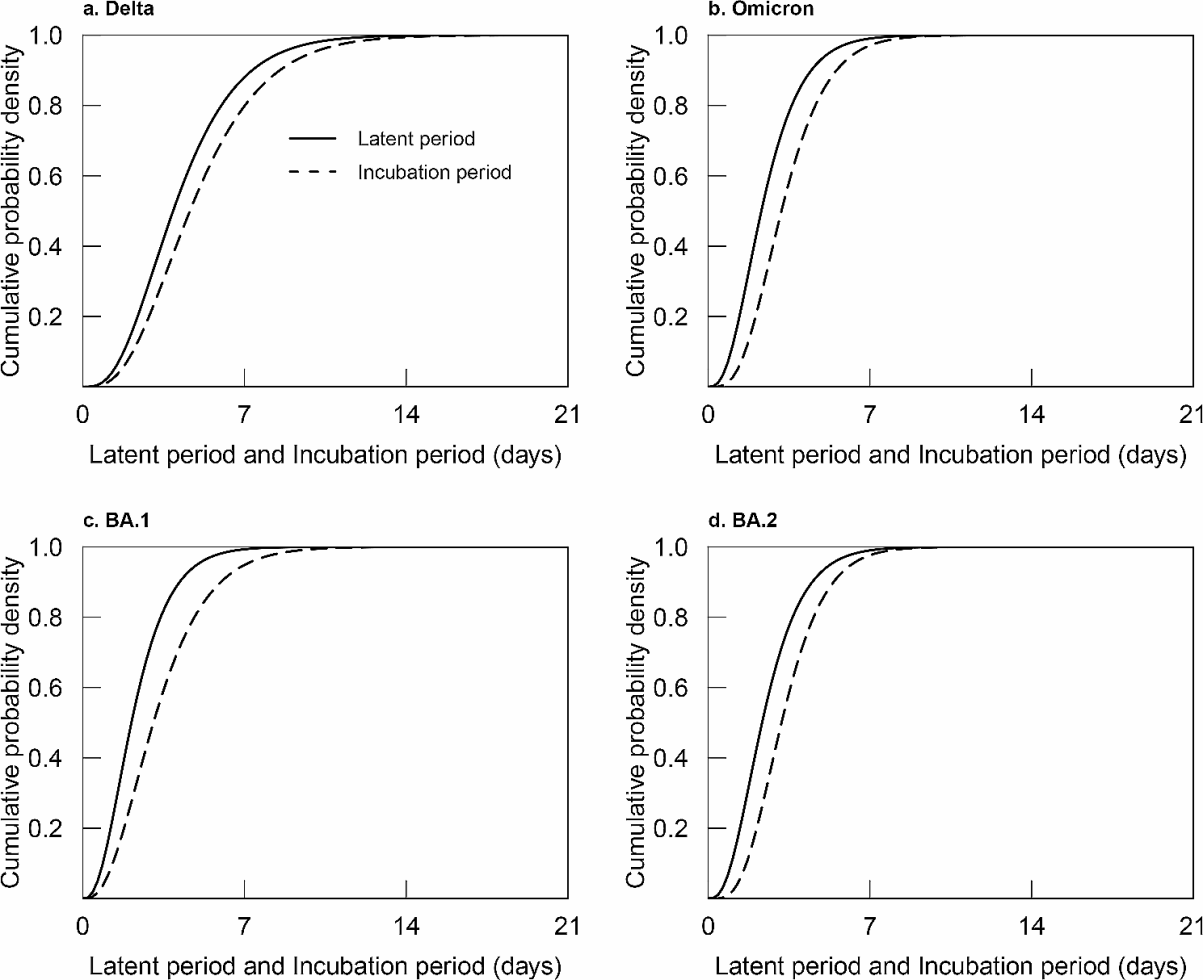
Cumulative probability density distributions of latent and incubation periods for symptomatic SARS-CoV-2 cases

In symptomatic Delta, Omicron, BA.1, and BA.2 cases, the mean latent period was 0.76, 0.85, 1.07, and 0.79 days shorter than the mean incubation period, respectively. The cumulative probability density distributions of latent and incubation periods for symptomatic cases are shown in Fig. 2.

Compared with incubation period, latent period was significantly shorter by 12% [exp($\beta$) = 0.88, 95%CI: 0.81~0.95, *p* = 0.001], 21%[exp($\beta$) = 0.79, 95%CI: 0.74~0.84, *p* < 0.001], 23% [exp($\beta$) = 0.77, 95%CI: 0.64~0.92, *p* = 0.004], and 20% [exp($\beta$) = 0.80, 95%CI: 0.74~0.86, *p* < 0.001] in the Delta, Omicron, BA.1, and BA.2 groups, respectively, after adjusting for age, number of infectors, exposure window, and shedding window.

## Discussion

In this study, we estimated and compared the latent and incubation periods of Omicron BA.1, BA.2, and Delta variants using a large sample of cases from 10 local epidemics in China. The latent and incubation periods of Omicron were shorter than those of Delta. Analyses using the AFT models revealed that age might be the main factor influencing the length of the latent period and incubation periods of both Omicron and Delta variants. After controlling for sex, vaccination status, clinical severity, etc., the latent period and incubation periods were longer in minors (0 ~ 17 years) and older individuals (≥ 50 years) compared with those aged 18 ~ 49 years.

Previous studies have shown that the mean latent period estimated based on censored data was gradually shortened in the wild-type strain (5.5 days) [4], the Delta variant (3.9 days) [5], and the Omicron variant (2.65~3.1 days) [2, 22]. This study revealed that 98.63% of Omicron patients started shedding virus within 7 days after exposure, consistent with Xin et al.’s (98.2%) results [2]. The mean incubation period for Omicron cases in this study was similar to a meta-analysis (3.42 days) [23], which included 142 studies with a total of 8112 study subjects between December 1, 2019 and February 10, 2022, also showing that the incubation period was gradually shortened in the wild-type strain, Delta variant, and Omicron variant. However, no statistically significant differences were observed in the incubation periods of BA.1 and BA.2 variants, consistent with another meta-analysis [24].

Pathogenetic studies have shown that the live viruses could be isolated from upper respiratory specimens of patients infected with Delta and Omicron variants 1 ~ 2 days before the onset of symptoms [1, 25]. This study showed that the mean latent period was 0.76 and 0.85 days shorter than the mean incubation period in symptomatic Delta and Omicron cases, respectively. Previous studies based on censored data have also shown that the differences between latent period and incubation period in the wild-type strain, Delta, and Omicron variants were not more than 2 days [2, 4, 5, 22]. Moreover, the latent period was significantly shorter than the incubation period in both Omicron and Delta variants after adjusting for possible confounding factors based on the AFT model. Therefore, although transmission occurred during the incubation period, the time of infectivity onset might be closer to the symptom onset for SARS-CoV-2 cases.

The incubation period often depends on the sensitivity of the immune system in cases. The stronger one’s immune response to respiratory viruses, the shorter the incubation period [26]. The longer COVID-19 incubation period observed in this study for older adults (≥ 50 years) we found may be due to the blunted immune response due either to age-associated immune senescence or secondary immunodeficiency in old age [8]. Older adults have a significantly higher proportion of highly differentiated effector and memory T cells due to their lifetime exposure to a variety of pathogens [27]. A significant proportion of T cells in the elderly have lost the ability to express costimulatory molecules [27–29] which may lead to a delay in the transmission of defense information by the immune system. Additionally, elderly people are likely to have recall bias and more underlying basic illnesses, so they more readily ignore early symptoms and only present for treatment when symptoms become more severe or intolerable. Accordingly, the time of symptom onset was erroneously assumed to have appeared later in the disease stage. In addition, a meta-analysis of a large sample size also showed that the mean incubation period of SARS-CoV-2 in people under 18 years of age was higher than that of the general population (*p* < 0.001) [23]. Research by the University of Hong Kong showed that reduced ${\beta }$-coronavirus immunity and T cell activation in children might cause milder COVID-19 pathogenesis [30]. Usually, children do not express the classical symptoms accurately, and this might need to be relayed by others, resulting in delayed reporting of the onset of symptoms [31]. Our previous research on the temporal distribution of positive RT-PCR results of imported SARS-CoV-2 infected individuals in China from July 24, 2020 to July 23, 2021 showed that the proportion of minors (≤ 18 years) and older adults (≥ 60 years) who were first RT-PCR positive after 14 days of entry was significantly higher than that of those who were first RT-PCR positive within 14 days of entry (*p* = 0.02) [32], suggesting that the latent period might be longer in minors and elders.

This study did not find significant differences in latent or incubation periods both between the Delta and Omicron groups with different vaccination doses. Ma et al [33] and Galmiche et al [13] reported comparable findings in individuals infected with the SARS-CoV-2 variant. Virological or immunological studies on the incubation and latent periods in relation to the level of vaccine immunization are still lacking. From a vaccine perspective, the protective efficacy varies with the vaccine type [34]. However, 98.98% (777/785 cases) and 91.18% (186/204 cases) of Omicron and Delta infected individuals had been vaccinated with the inactivated vaccine in this study. Furthermore, some studies have shown that sequential vaccination is more effective, [35] but our study only involved individuals immunized with a homologous booster. Whether vaccination has an effect on the latent period or the incubation period needs to be more explored.

In the early stages of the epidemic, studies showed that infected individuals with shorter incubation periods were more likely to have severe disease and a longer disease course. Cai et al.^[10]^ showed that patients with a shorter incubation period (≤ 7 days) developed more severe illness, had longer hospital stays, and the time intervals between symptom onset and discharge were longer than those with a longer incubation period (> 7 days). Lai et al [11] and Huang et al [12] also suggested that a shorter incubation period was associated with the severe progression of COVID-19 disease. We found that the latent period decreased progressively along asymptomatic or mild, moderate, severe, or critical illness groups among Delta cases aged ≥ 50 years, and the incubation period also decreased progressively from moderate to severe or critical infections. Although these differences were not statistically significant (**Additional file 2: Table S4**). In this study, most cases had predominantly mild and moderate illnesses, with severe (and critical) illnesses accounting for only 3.13% (21/672) and 0.11% (1/885), respectively, of Delta and Omicron cases. The extent of analyses for clinical severities was greatly limited by the sample size. The Omicron variant had a reduced ability to replicate in the lung compared with previous strains [36–38]. Accordingly, it is therefore less likely to cause severe disease after infection. Meanwhile, it is essential to obtain a sufficient number of severe or critical cases to fully explore the impact of clinical severity on the latent and incubation periods.

There were some limitations to this study. First, there might be a bias in the sample selection process. For example, Omicron cases were predominant in young adults, with 16.61% (147/885 cases) in the age group 0 ~ 17 years and 7.46% (66/885 cases) in the age group ≥ 65 years. Thus, caution is needed when extrapolating the findings or comparing our findings with the results of other studies. Second, we used the time interval between infection and the first date of the positive RT-PCR test (cycle threshold < 40) to estimate the latent period. However, there is a possibility that transmission will not occur until the viral load reaches a certain level. Thus, our approach might underestimate the latent period [2]. Third, due to the large sample size of “unknown”, we only did a univariate analysis for the comorbidity, the stability of the association between the comorbidity and latent as well as the incubation periods was limited. Fourth, we fitted the parametric model by choosing the best fit among the Lognormal, Gamma, and Weibull distributions only, and we did not consider all possible model distributions. In addition, despite the longitudinal and in-depth investigation of each case and its contacts, we could not completely avoid recall bias in the individual records. However, with the use of a combination of “Big Data” to track the travel trajectories of those involved in the epidemic and symptom monitoring during the medical observation period to obtain epidemiological investigation information in this study, the possibility of our recall bias was largely reduced.

## Conclusion

Pre-symptomatic transmission can occur in Delta, BA.1, and BA.2 cases. The latent and incubation periods between BA.1 and BA.2 were similar but shorter compared with Delta. Age may be associated with the latent and incubation periods of SARS-CoV-2.

### Electronic supplementary material

Below is the link to the electronic supplementary material.

**Supplementary Material 1:** Summary of supplementary information

**Supplementary Material 2:** Additional file 1

**Supplementary Material 3:** Additional file 2

## Data Availability

The data supporting the findings of this study are available in the manuscript or in the Additional Files. Further requests might require ethical approval and should be made to the corresponding author.
