# Supplementary material for: Latent and incubation periods of Delta, BA.1, and BA.2 variant cases and associated factors: a cross-sectional study in China
Source: BMC Infect Dis. 2024 Mar 6;24:294. doi: 10.1186/s12879-024-09158-7 (PMC10916204; doi:10.1186/s12879-024-09158-7)
Supplement: Supplementary file 1 — Supplementary Material 1: Summary of supplementary information [file 12879_2024_9158_MOESM1_ESM.docx]

**Supplementary information**

Manuscript title: Latent and incubation periods of Delta, BA.1, and BA.2 variant cases and associated factors: a cross-sectional study in China

**Additional file 1: Details of the methods.**

**Additional file 2: Table S1.** Akaike Information Criterion (AIC) of latent and incubation distributions with different models. A lower AIC indicates a better model fit. **Table S2.** Sensitivity analysis of the three treatments in which the$V_{L}$ was unclear or before the $E_{L}$ in parametric estimates of latent period. **Table S3.** Association between selected factors and latent as well as incubation periods using the multivariate AFT model. **Table S4.** Association between clinical severity and latent as well as incubation periods of Delta variant in cases aged ≥50 years.
