# Supplementary material for: Latent and incubation periods of Delta, BA.1, and BA.2 variant cases and associated factors: a cross-sectional study in China
Source: BMC Infect Dis. 2024 Mar 6;24:294. doi: 10.1186/s12879-024-09158-7 (PMC10916204; doi:10.1186/s12879-024-09158-7)
Supplement: Supplementary file 2 — Supplementary Material 2: Additional file 1 [file 12879_2024_9158_MOESM2_ESM.docx]

**Additional file 1**

Manuscript title: Latent and incubation periods of Delta, BA.1, and BA.2 variant cases and associated factors: a cross-sectional study in China

**Definitions of clinical severity and vaccination history**

We classified SARS-CoV-2 infected individuals as asymptomatic, mild, moderate, severe and critical according to the COVID-19 Diagnostic and Therapeutic Guidelines (9^th^ version) published by National Health Commission since 14 March 2022 [1] to assess their clinical severity. Asymptomatic infected individuals were cases who tested positive for SARS-CoV-2 but did not have any symptoms that might be associated with COVID-19, such as fever, dry cough, fatigue, nasal congestion, runny nose, dry throat, sore throat, loss of taste or smell, conjunctivitis, myalgia, diarrhea, etc., and did not have an evidence of pneumonia on imaging. Mild infected individuals were those with mild symptoms and no evidence of pneumonia on imaging. Moderate infected individuals were those with the symptoms associated with COVID-19, and pneumonia on imaging. Cases were defined as severe if adults met one of the following criteria: (1). Shortness of breath, RR ≥30 breaths/min; (2). Oxygen saturation ≤93% on inspired air at rest; (3). PaO2/FiO2 ≤300 mmHg (1 mmHg = 0.133 kPa); (4). Progressive exacerbation of clinical symptoms with marked progression of >50% of the lesion on lung imaging within 24 to 48 hours. If children met any of the following: (1). Persistent high fever for more than 3 days; (2). Presence of shortness of breath (<2 months of age, RR ≥60 breaths /min; 2-12 months of age, RR ≥50 breaths /min; 1-5 years of age, RR ≥40 breaths /min; >5 years of age, RR ≥30 breaths /min), except for the effects of fever and crying; (3). Fingerprinted oxygen saturation of ≤93% on air inhalation at rest; (4). Assisted breathing (Nasal flaring, triple concave sign); (5). Presence of lethargy and convulsions; (6). Refusal of food or feeding difficulties with signs of dehydration. Cases were defined as critical if they met one of the following criteria: (1). Respiratory failure requiring mechanical ventilation; (2). Shock; (3). Comorbid other organ failure requiring intensive care unit.

In our study, at least fully vaccinated cases were defined as cases in which the midpoint of the exposure window was more than 14 days after the 2^nd^ dose of inactivated vaccine, the 3^rd^ dose of recombinant protein vaccine, or the 1^st^ dose of adenovirus-vectored vaccine. Cases were defined as receiving booster vaccination series if the midpoint of the exposure window was more than 14 days after the 3^rd^ dose of vaccine (inactivated or adenovirus-vectored or recombinant protein vaccine) for those who were at least fully vaccinated with inactivated vaccine, or more than 14 days after the 2^nd^ dose of adenovirus-vectored vaccine for those who were at least fully vaccinated with adenovirus-vectored vaccine. Fully vaccinated cases met the criteria for “at least fully vaccinated” but not “booster vaccination”. Partially vaccinated cases were those who had been vaccinated but did not meet the criteria for “at least fully vaccinated”.

**Statistical analysis**

**Parametric models accounted for interval-censoring**

We need to account for the interval-censoring on exposure window and shedding window when estimating latent period and incubation period using the maximum likelihood method.[2] We assumed $f_{latent}(.)$ and $f_{incubation}(.)$ follow Gamma, Lognormal, or Weibull distribution. The likelihood functions were constructed as follows:

$$L_{latent}=\int_{E_{L}}^{E_{U}} \int_{max(E,V_{L})}^{V_{U}} f_{latent}(V-E)dVdE$$

$$L_{incubation}=\int_{E_{L}}^{E_{U}} f_{incubation}(S-E)dE$$

S was defined as symptom onset. The best-fitted model was determined by the smallest value of Akaike’s Information Criterion. The Gamma model was finally selected to estimate the latent and incubation periods with the smallest Akaike’s Information Criterion values (**Additional file 2: Table S1**). The mean, standard deviation (SD), median, and other percentiles were derived from the Gamma model.

$$f\left( x \right)=\frac{1}{\theta^{k}\Gamma\left( k \right)}x^{k-1}e^{-\frac{x}{\theta}} x,k,\theta>0$$

**Analysis of factors associated with latent and incubation periods**

Accelerated failure time (AFT) model is a liner regression model used to analyze factors associated with interval censored data [3,4]:

$$logT_{i}=\mu+\beta_{1}x_{1}+\ldots+\beta_{p}x_{p}+\sigma\varepsilon_{i}$$

$logT_{i}$ indicates the log-transformed latent period or incubation period，while $x_{1},\ldots x_{p}$ represent the factors associated with coefficients $\beta_{1},\ldots\beta_{p}$. $\varepsilon_{i}$ is the residual after logarithmic transformation. The estimated regression coefficients, $\beta$, can be interpreted as the expected change in median log latent period or incubation period relative to baseline, while the transformed effects exp ($\beta$) can be interpreted as acceleration factors, and proportional increases or decreases in the median latent or incubation periods.

**Compare the latent period and incubation period**

The AFT model was used to test the significance of the difference between the latent period and the incubation period. The latent period and incubation period data were combined, log-transformed to form $logT_{i}$. The independent variable “data type” was added, which was divided into “latent period” and “incubation period”.

**References**

[1] NHCPRC. Guidelines in Diagnosis and Treatment of COVID-19 (ninth version). 2022. <https://www.gov.cn/zhengce/zhengceku/2022-03/15/5679257/files/49854a49c7004f4ea9e622f3f2c568d8.pdf> (accessed 20 March 2022).

[2] Xin H, Li Y, Wu P, et al. Estimating the Latent Period of Coronavirus Disease 2019 (COVID-19). *Clin Infect Dis* 2022; **74**(9): 1678-81. <https://doi.org/10.1093/cid/ciab746>.

[3] Cowling BJ, Muller MP, Wong IO, et al. Alternative methods of estimating an incubation distribution: examples from severe acute respiratory syndrome. *Epidemiology* 2007; **18**(2): 253-9. <https://doi.org/10.1097/01.ede.0000254660.07942.fb>.

[4] Zhang ZG, Sun JG. Interval censoring. *Statistical Methods in Medical Research* 2009; **19**(1): 53-70. <https://doi.org/10.1177/0962280209105023>.
