# Supplementary material for: Latent and incubation periods of Delta, BA.1, and BA.2 variant cases and associated factors: a cross-sectional study in China
Source: BMC Infect Dis. 2024 Mar 6;24:294. doi: 10.1186/s12879-024-09158-7 (PMC10916204; doi:10.1186/s12879-024-09158-7)
Supplement: Supplementary file 3 — Supplementary Material 3: Additional file 2 [file 12879_2024_9158_MOESM3_ESM.docx]

**Additional file 2**

Manuscript title: Latent and incubation periods of Delta, BA.1, and BA.2 variant cases and associated factors: a cross-sectional study in China

**Table S1.** Akaike Information Criterion (AIC) of latent and incubation distributions with different models. A lower AIC indicates a better model fit.

|  | **Latent period** | |  | **Incubation Period** | |
| --- | --- | --- | --- | --- | --- |
|  | **Omicron (885)** | **Delta (672)** |  | **Omicron (420)** | **Delta (405)** |
| **Lognormal** | 1056.210 | 381.227 |  | 958.044 | 1061.432 |
| **Gamma** | 1032.456 | 364.705 |  | 941.104 | 1051.301 |
| **Weibull** | 1032.466 | 365.137 |  | 947.127 | 1058.692 |

**Table S2.** Sensitivity analysis of the three treatments in which the$V_{L}$ was unclear or before the $E_{L}$ in parametric estimates of latent period.

|  | **Omicron (885)** | | | |  | **Delta (672)** | | | |
| --- | --- | --- | --- | --- | --- | --- | --- | --- | --- |
|  | **Mean** | **SD** | **95th percentile** | **99th percentile** |  | **Mean** | **SD** | **95th percentile** | **99th percentile** |
| $\boldsymbol{E}_{\boldsymbol{L}}$**+0 day** | 2.57 | 1.54 | 5.51 | 7.41 |  | 4.40 | 2.47 | 9.08 | 12.05 |
| $\boldsymbol{E}_{\boldsymbol{L}}$**+0.5 days** | 2.58 | 1.52 | 5.48 | 7.36 |  | 4.40 | 2.46 | 9.07 | 12.03 |
| $\boldsymbol{E}_{\boldsymbol{L}}$**+0.9 days** | 2.59 | 1.49 | 5.43 | 7.24 |  | 4.41 | 2.45 | 9.04 | 11.97 |

**Table S3.** Association between selected factors and latent as well as incubation periods using the multivariate AFT model.

|  | **Latent period** | | |  | **Incubation period** | | |
| --- | --- | --- | --- | --- | --- | --- | --- |
|  | **N** | **exp (**$\boldsymbol{\beta}$**) (95%*CI*)** | **p value** |  | **N** | **exp (**$\boldsymbol{\beta}$**) (95%*CI*)** | **p value** |
| **Variants** |  |  |  |  |  |  |  |
| Delta | 672 | 1 (ref) | .. |  | 405 | 1 (ref) | .. |
| Omicron | 885 | 0.60 (0.53~0.67) | <0.001 |  | 420 | 0.74 (0.65~0.84) | <0.001 |
| **Sex** |  |  |  |  |  |  |  |
| Male | 725 | 1 (ref) | .. |  | 354 | 1 (ref) | .. |
| Female | 832 | 0.98 (0.91~1.05) | 0.569 |  | 471 | 0.98 (0.91~1.05) | 0.534 |
| **Age** |  |  |  |  |  |  |  |
| 0~17 | 243 | 1 (ref) | .. |  | 122 | 1 (ref) | .. |
| 18~33 | 442 | 0.79 (0.70~0.88) | <0.001 |  | 247 | 0.75 (0.66~0.85) | <0.001 |
| 34~49 | 340 | 0.82 (0.72~0.93) | 0.003 |  | 191 | 0.81 (0.70~0.93) | 0.003 |
| 50~65 | 303 | 1.03 (0.91~1.17) | 0.650 |  | 148 | 0.95 (0.82~1.10) | 0.485 |
| 66~ | 229 | 1.13 (0.99~1.29) | 0.074 |  | 117 | 0.98 (0.84~1.15) | 0.830 |
| **Clinical severity** |  |  |  |  |  |  |  |
| Asymptomatic | 166 | 1 (ref) | .. |  | NA | NA | NA |
| Mild | 743 | 1.04 (0.92~1.16) | 0.559 |  | 435 | 1 (ref) | .. |
| Moderate/Severe/Critical | 648 | 0.99 (0.86~1.14) | 0.922 |  | 390 | 1.03 (0.92~1.15) | 0.636 |
| **Vaccination history** |  |  |  |  |  |  |  |
| Unvaccinated | 359 | 1 (ref) | .. |  | 207 | 1 (ref) | .. |
| Partially vaccinated | 209 | 1.00 (0.89~1.12) | 0.964 |  | 131 | 0.96 (0.85~1.08) | 0.460 |
| Fully vaccinated | 566 | 1.04 (0.93~1.16) | 0.459 |  | 289 | 1.00 (0.89~1.12) | 0.986 |
| Booster | 423 | 0.96 (0.84~1.11) | 0.604 |  | 198 | 1.02 (0.88~1.18) | 0.793 |
| **Number of infectors** |  |  |  |  |  |  |  |
| >1 | 681 | 1 (ref) | .. |  | 330 | 1 (ref) | .. |
| =1 | 876 | 0.96 (0.89~1.03) | 0.280 |  | 495 | 0.98 (0.91~1.06) | 0.609 |
| **Exposure window** |  |  |  |  |  |  |  |
| ≤3 days | 927 | 1 (ref) | .. |  | 481 | 1 (ref) | .. |
| >3 days | 630 | 1.26 (1.16~1.36) | <0.001 |  | 344 | 1.24 (1.14~1.35) | <0.001 |
| **Shedding window** |  |  |  |  |  |  |  |
| ≤3 days | 1100 | 1 (ref) | .. |  | 548 | 1 (ref) | .. |
| >3 days | 457 | 0.81 (0.74~0.90) | <0.001 |  | 277 | 1.06 (0.98~1.15) | 0.157 |

Notes: NA represented that the figure was not applicable for this cell.

**Table S4.** Association between clinical severity and latent as well as incubation periods of Delta variant in cases aged ≥50 years.

|  | **Latent period** | | |  | **Incubation period** | | |
| --- | --- | --- | --- | --- | --- | --- | --- |
|  | **N** | **exp (**$\boldsymbol{\beta}$**) (95%*CI*)** | **p value** |  | **N** | **exp (**$\boldsymbol{\beta}$**) (95%*CI*)** | **p value** |
| Asymptomatic/Mild | 20 | 1 (ref) | .. |  | 11 | 1 (ref) | .. |
| Moderate | 287 | 0.85 (0.64~1.11) | 0.225 |  | 166 | 1.02 (0.72~1.45) | 0.900 |
| Severe/Critical | 21 | 0.71 (0.46~1.09) | 0.118 |  | 13 | 0.73 (0.45~1.17) | 0.186 |

Notes: Adjusted for exposure window, shedding window and number of infectors.
